# Evaluation of photodynamic therapy in pericoronitis: Protocol of randomized, controlled, double-blind study

**DOI:** 10.1097/MD.0000000000015312

**Published:** 2019-04-26

**Authors:** Tânia Oppido Schalch, Michelle Palmieri, Priscila Larcher Longo, Paulo Henrique Braz-Silva, Isabel Peixoto Tortamano, Edgard Michel-Crosato, Marcia Pinto Alves Mayer, Waldyr Antonio Jorge, Sandra Kalil Bussadori, Christiane Pavani, Renata Matalon Negreiros, Anna Carolina Ratto Tempestini Horliana

**Affiliations:** a Postgraduate Program in Biophotonics Applied to Health Sciences, University Nove de Julho, UNINOVE; b Department of Stomatology, School of Dentistry, University of São Paulo; c Aging Sciences Program; d Laboratory of Virology, Institute of Tropical Medicine of São Paulo; e Integrated Clinic, Department of Stomatology; f Community Dentistry Department, School of Dentistry; g Department of Microbiology, Institute of Biomedical Sciences, University of São Paulo.

**Keywords:** laser, microbiological analysis, pericoronitis, photodynamic therapy, randomized controlled clinical study, third molar

## Abstract

**Introduction:**

Pericoronitis is a common disease in the eruption phase of third molars, sometimes debilitating, with an impact on the quality of life. The most indicated treatment in the initial phase is the irrigation for cleanliness of the region. In order to reduce the chances of systemic dissemination of the infection and antibiotics use, it is mandatory to test effective treatments in the initial phase of pericoronitis avoiding the evolution of the infectious disease. Photodynamic therapy (PDT) is an interesting alternative because it is an effective antimicrobial treatment that is easy to perform and does not select bacterial resistance. The methylene blue (MB) used in PDT has been studied in an oral formulation, which optimizes the formation of monomers increasing its antimicrobial action.

**Objective:**

The aim of this study is to evaluate the effectiveness of PDT with MB in an astringent vehicle in pericoronitis on the initial phase in healthy patients through microbiological, clinical, and immune response. The impact of pericoronitis on oral health-related quality of life (OHRQoL) of these patients will also be evaluated.

**Method:**

In this randomized, controlled, double-blind clinical bioequivalence protocol, 64 healthy patients with pericoronitis will be evaluated. Patients will be randomized into the positive control group (G1) (n = 32): irrigation with sterile saline and PDT (conventional MB at 0.005% concentration and irradiation with low intensity laser λ = 660 nm, 9J per point and radiant exposure of 318 J/cm^2^), and the experimental group (G2) (n = 32): treatment identical to G1, however, MB will be delivered in a new formulation for oral use. Microbiological analysis will be performed by RT-PCR for the bacterium *Tannerella forsythia.* Gingival crevicular fluid and saliva will be collected to evaluate cytokines by Luminex assay (Luminex Corporation, Austin, TX). The pain (visual analogue scale), swelling and buccal opening (digital caliper), and OHRQoL will also be evaluated through the OHIP-14 questionnaire. The variables will be evaluated in T1 (baseline), T2 (immediately after PDT), and T3 (4th day after PDT). Registration: clinicaltrials.gov NCT 03576105. Registered in July 2018.

## 1 Introduction

In order to reduce the chances of systemic dissemination of the infection and the use of antibiotics, it is necessary to test effective alternative treatments in the initial phase of pericoronitis and also avoid the evolution of local infectious. Photodynamic therapy (PDT) is an interesting alternative because it is an effective antimicrobial treatment that is easy to perform and does not select bacterial resistance.^[1–3]^ The PDT also has been widely used for decontamination of endodontic channels, periodontal pockets, and reduces halitosis in adolescents, and has been considered as a noninvasive alternative and an efficient antimicrobial therapeutic method.^[3–5]^

PDT is used for decontamination, microbial reduction, in which a photosensitizer (PS) is activated by light at a specific wavelength (λ = 660 nm).^[6,7]^ The absorption of the photons by the PS, leads to a state of triple excitation of the oxygen resulting in energy or transference of the electron to the molecule of oxygen, with consequent formation of reactive species, like singlet oxygen.^[8]^

PDT relies on the appropriate combination of light, PS, and oxygen.^[9]^ Light reach the PS at the target cell in the presence of oxygen, leading to PS excitation.^[10]^ The singlet excited state PS molecules can be converted to a triplet excited state by intersystem crossing.^[2,11,12]^ The PS triplet excited state transfer energy (type II reaction) or electron (type I reaction) to the oxygen molecule, with consequent formation of singlet oxygen or free radicals, respectively.^[2,7,8,10]^ These oxidant species lead to toxic and destructive action in bacteria.^[13]^

The PS can be selectively incorporated into the bacterium.^[2,10]^ This PS selectivity may be related to time of PS action before irradiating.^[12]^ The antimicrobial effect is based on the action of the reactive oxygen species (ROS) on bacterial membrane, organelles, and DNA.^[12,10]^

Methylene blue (MB) is a phenothiazinium, cationic dye that absorbs red light.^[6]^ It is commonly used as PS for antimicrobial action at concentrations of 0.005% to 0.01%.^[12,14,15]^

Depending on the concentration, MB ground state may also work as an electric donor allowing electron transfer reactions that can either destroy PS or allow formation of other ROS, ionic strength, temperature, media, and interaction with target tissue molecules, MB can bind to other molecules forming aggregation and dimer, factor that will influence the reactions type I or type II described before.^[2,11]^ Its participation in the photosensitization process can interfere with the antimicrobial potential of PDT.^[10,16]^

PDT have additional beneficial effects at clinical and immunological aspects, because it is also responsible for photobiomodulation in tissues.^[12]^ The mechanism occurs by apoptosis, acting specifically on the mitochondria and the lysosome, since light acts improving tissue repair and decreasing the periodontal inflammatory process, that will modulate the immunoinflammatory response of the host, interfering in the levels of inflammatory cytokines.^[2,13,17]^

Due to the role of PDT and the need for new treatments for pericoronitis, the aim of this study is to evaluate the effectiveness of PDT with MB in an oral formulation in pericoronitis in the initial phase in healthy patients through microbiological, clinical, and immune response. The impact of pericoronitis on oral health-related quality of life (OHRQoL) of these patients will also be evaluated.

## 2 Methods

The protocol was approved by the Research Ethics Committee of Nove de Julho University (UNINOVE), number # 2.732.106 and registered at clinicaltrials.gov NCT 03576105. The study is in accordance with the Declaration of Helsinki (revised in Fortaleza, 2013).

Participants who accept to participate will sign the Informed written Consent Form after verbal and written explanation of the study. The sample will comprise 64 healthy patients of both genders aged from 13 to 60 with pericoronitis. After agreeing to participate, in a bio-equivalence study, participants will be evaluated in 3 different moments: baseline before PDT (T1), immediately after PDT (T2), and 4th day after PDT (T3). The clinical evaluation and the PDT treatment will be performed by the same researcher, in all 3 moments at the Dental Clinic at Nove de Julho University—UNINOVE, São Paulo, Brazil, from March, 2019 until September 2019.

### 2.1 Sample size calculation

The sample size was calculated (G∗ Power software version 3.1.9.2, Kiel, Germany) by *t* test for paired groups, since we have 2 groups (G1 conventional PS and G2 oral use PS). The effect size was determined using the formula: 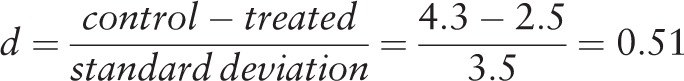


We select the worst scenario, that is, the largest standard deviation between means. The mean values of control and treated groups, as well as the standard deviation were taken from 1 study.^[18]^ The error was set at 5% and the power test at 95%. This value was calculated to provide 95% strength (α = 0.05). According to the calculus, a ample of 64 patients will be necessary to detect differences in *Tannerella forsythia* level.^[18]^ Other factors related to secondary outcomes will be analyzed statistically.

### 2.2 Inclusion and exclusion criteria

Participants in this study will be evaluated to meet the eligibility criteria: ASA I patients (negative medical history), with age of 13 years old or over till 60 years, with lower third molar erupted or partially erupted, with pericoronitis. Patients should have at least one lower third molar partially visible in the oral cavity to be examined. All participants, regardless of age, gender, cultural level, or socioeconomic status may participate in the study. The following patients will be excluded: those allergic to MB, pregnant or breastfeeding women, with presence of purulent exudate, those who have used anti-inflammatory drugs or antibiotic medications for the last 3 months, those with fever (with temperature above 37.8°C), that may suggest an infection. The local temperature will be measured in the mandibular angle region 2 cm above the lower jaw board (Safety 1st model “No Touch Forehead,” Columbus, OH).

### 2.3 Randomization

An external researcher who will not participate in this study will perform randomization through the Microsoft Excel program, version 2013 (Microsof, Redmont, WA). To decide the type of the PS, another researcher will randomize into 4 blocks with 16 patients blocking in pairs of numbers (1:1) designed as A or B. As the study will be performed in a double-blind manner, neither the patient nor the operator will know which MB will be used as PS. To randomly distribute the treatments, the same researcher (not involved in the study) will prepare the envelopes. The letters (A or B) drawn will be placed into opaque envelopes labeled with sequential numbers. The envelopes will be sealed and remain in the same numerical order in a safe place until PDT treatment. These data will only be revealed after statistical analysis.

### 2.4 Design

This is a controlled, randomized, double-blind, clinical trial:

- G1—(positive control group) 32 patients with pericoronitis, irrigated with sterile saline and the PDT performed with conventional MB. The irradiation will be performed with low intensity laser λ = 660 nm, 9J per point and radiant exposure of 318 J/cm^2^.
- G2—(experimental group) 32 patients with pericoronitis treatment identical to G1, however the PDT will be delivered in a new formulation for oral use (patent application INPI BR1020170253902).^[19]^

### 2.5 Methodology for irradiation: low level laser specifications and dosimetry

All participants (G1 and G2) will be irrigated with 20 mL of sterile saline in the gingival sulcus of the lower third molar. Before irradiating 0.4 mL of blue solution, at the concentration of 0.005% will be applicated, which may be conventional MB or a new formula for oral use.^[19]^ As the MB has the function of PS, it will be applied inside the gingival sulcus around the third molar with pericoronitis after 3 minutes, and the irradiation will be initialized.

**Figure 1:**
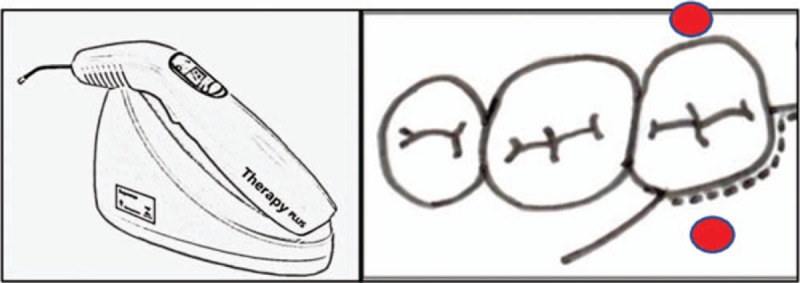
Irradiation points.

The red laser diode (Therapy XT DMC, São Carlos, Brazil) (ANVISA 80030810157) with a wavelength of 660 nm (± 10 nm) will be used. The power of the appliance is 100mW. The diameter of the fiber optic device is 600 μm and thus, a spot (area) of 0.002826 cm^2^. It will deliver the energy of 9 J per point in 90 seconds, generating Radiant Exposure (fluence) of 318 J/cm^2^ and irradiance will be 31.8 W/cm^2^. The irradiance will be 3.5W/cm^2^. Participants will receive irradiation at 2 points: 1 buccal and 1 lingual (Fig. 1). During application the volunteer and operator will wear goggles, the active tip of the instrument will be protected with disposable transparent plastic adapted to tip to prevent cross-infection, and the operator will be putting on protective gloves.

### 2.6 Outcome measures

All the variables (except *T forsythia* and cytokines) described below will be evaluated by the same operator at baseline, before PDT (T1), immediately after PDT (T2), and on the 4th day after PDT (T3). *T forsythia,* salivary and crevicular cytokines will be evaluated at T1 and T3.

- The primary outcome will be the presence and quantity of bacterium *T forsythia.* Microbiological analysis will be performed by RT-PCR for the bacterium *T forsythia*. Biofilm samples for microbiological analysis, will be collected at 2 points of the vestibular gingival sulcus of the lower third molar using Gracey mifive curettes (Hu-Friedy, Chicago). The collected samples will be maintained in TE (Tris-EDTA), conditioned in microtubes in ice, and later transported to the laboratory analysis in the Microbiology Laboratory of ICB-USP Department of Microbiology, Institute of Biomedical Sciences for laboratory analysis. From these samples, total DNA will be extracted with Step One Plus Thermo-Time PCR System (Applied Biosystem, Foster City, CA) before amplificating them with PCR. The products will be detected by fluorescence using the Quantimix Easy SYG Kit (Biotools, Madrid, Spain), according manufacturer. For the reaction, 10 μL of SYBR Green, 0.5 μL DNA template, 200 mM of each primer corresponding to the gene (described in Table 1) will be used. Amplicons from conventional PCR will be used as quantify control curve.

**Table 1:**
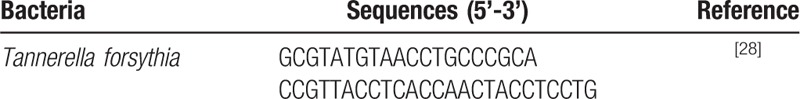
Sequences and reference of the primers.

Secondary outcomes will be pain, swelling, trismus, profile of clevicular cytokines, OHRQoL, and position and classification of the lower third molars.

**Figure 2:**
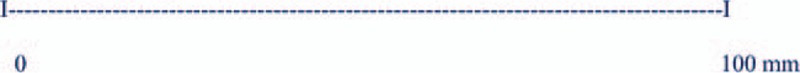
Visual analog scale (VAS).

**Figure 3:**
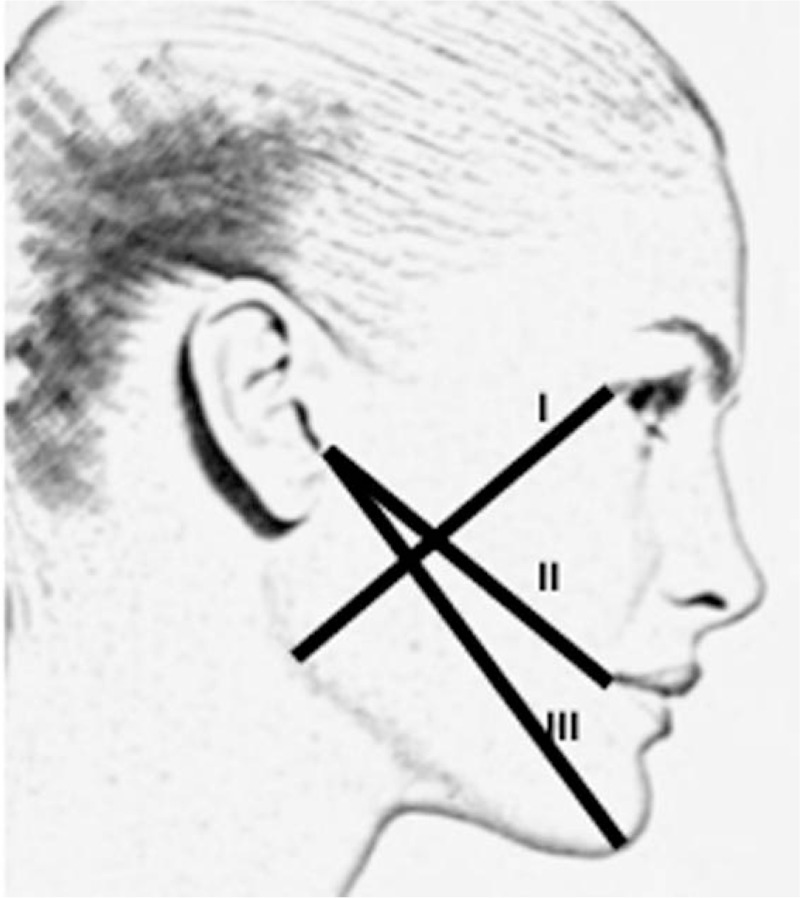
Three pre-established facial measures. The criteria for the determination of edema—(I) corner of the eye to angle of the jaw, (II) tragus to the labial commissure, and (III) tragus to pogonion.

- The pain will be assessed by applying visual analog scale (VAS), consisting of a 100-mm line numbered in centimeters, with 2 closed ends. One end is labeled “0” and the other “100,” meaning no pain and terrible pain, respectively (Fig. 2). Each patient will be instructed to mark a vertical line on the point that best matches the intensity of pain during the evaluation. Instructions on marking will always be given to the patient by the same operator.^[20]^
- The criteria for the determination of swelling will follow pre-established measurements in the face: corner of the eye to angle of the jaw, tragus to the labial commissure, and tragus to pogonion^[21]^ (Fig. 3). The edema value will be the sum of the 3 pre-established facial measures (using a digital caliper).^[23]^
- For evaluation of the presence of trismus the inter-incisor will be measured (distance between the incisal edge of the maxillary central incisor and the lower central incisor) will be measured with a digital caliper to determine the buccal opening (Mitutoyo Digimatic Caliper model, Japan).
- The profile of gingival crevicular fluid (GCF) and salivary cytokines will be evaluated and should be collected before the biofilm collection. It will be assessed by using 10 mL of non-stimulated saliva, kept in ice cubes tubes and sent to the Laboratory of Virology, Institute of Tropical Medicine of São Paulo, University of São Paulo, for further analysis. The GCF will be collected from the mesiobuccal portion of lower third molar in each patient,^[23]^ before PDT (T1) and 4 days post PDT (T3) in both groups. Before collection, the supragingival plaque will be removed using a sterile instrument. The site will be insulated using cotton rolls and dried using a small jet of air (not in the groove/pocket). A sterile paper strip into a microcentrifuge tube will be inserted into the gingival sulcus until resistance. The strip will stay in place for 30 seconds. Strips contaminated with blood will be discarded and the site was resampled after 90 seconds. The GCF strips will be stored in microcentrifuge tubes at −80° C until analysis.^[23]^ The cytokines (interleukin [IL]-1β, IL-6, IL-10, and tumor necrosis factor alpha [TNFα]) from the GCF will be analyzed by high-sensitivity human cytokine 4-plex by Millipore (Millipore Corporation, Billerica, MA). The Milliplex kits were developed with microspheres and were based on immunoassays.
- OHRQoL will be assessed by Brazilian version of OHIP-14 questionnaire.^[24]^ This instrument consists of 14 items arranged in 7 factors: functional limitation, physical pain, psychological discomfort, physical disability, psychological disability, social disability, and handicap. The answers are given corresponding to a total of 5 points on a Likert-type scale. The scale included the following responses: never (coded 0), hardly ever (coded 1), occasionally (coded 2), fairly often (coded 3), and very often (coded 4). The OHIP-14 scale ranged from 0 to 56, with higher scores indicating poorer QoL.^[25]^ The items of the OHIP-14 are shown in Table 2.
- Position and classification of the lower third molars will be assessed by panoramic radiography, according to the classification of Pell and Gregory and Winter,^[26]^ performed by only 1 evaluator, following the criteria of imaginary lines as proposed by Almendros-Marques et al.^[27]^

**Table 2:**
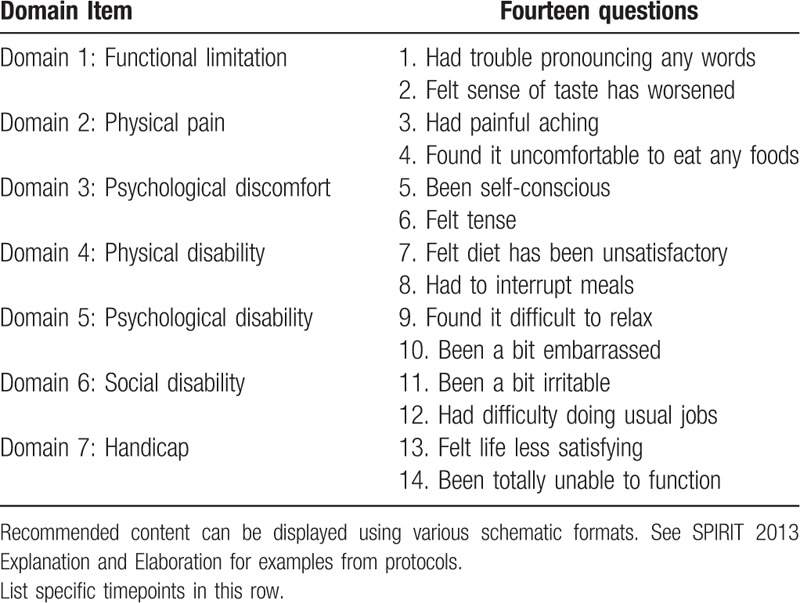
OHIP-14 questionnaire and its domains.

**Figure 4:**
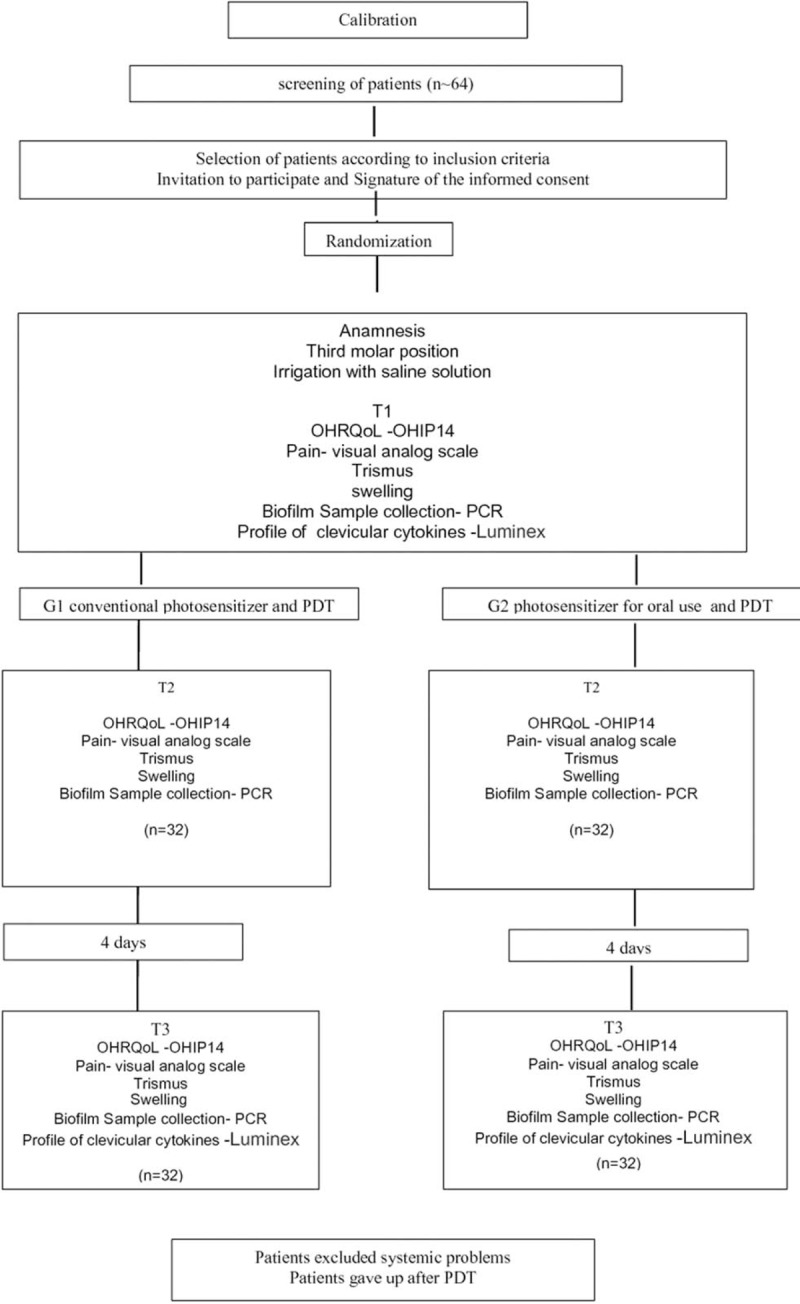
Flowchart presenting an overview of the study.

A flowchart is presented for an overview of the study (Fig. 4).

### 2.7 Statistical analysis

The data will be collected, tabulated on Excel table developed for research and presented in a descriptive way. All statistics will be conducted using the SPSS 21 program package (SPSS Inc, Chicago, IL). In the first part of the results will be performed the descriptive analysis. Measures of: absolute number, mean, standard deviation, minimum value and maximum value, percentage and cumulative percentage will be used. The bacterial count will be normalized by conversion into logarithmic values will be compared by the student *t* test. In the second part, we will verify the inferential analysis for the numerical variables. Tests of Normality Skewness and Kurtosis will be used, then verification of the homogeneity of the variables. The ANOVA will be applied for repeated data, and the Spearman correlation, and the Huynh-Feldt, Greenhouse-Geisser, Box's conservative tests. If there is no normality of the sample will be used The Wilcoxon test will be used in order to compare before and after the PDT. The *P* value is set at *P* < .05 (95% level of significance). By the end of the study, randomized groups will be revealed and statistical analysis will be performed as group A or B. The meaning of A or B will be known after statistical analysis. The data will be evaluated by comparing 2 groups: experimental and control groups (gold standard).

## 3 Discussion

The main rationale for conducting this study is to verify a possibility of treating pericoronitis with a low risk alternative. Other reasons are to suppress or reduce the quantity of antibiotics medications and to test if the new oral formulation with MB is more effective on bacterial reduction.

The primary outcome will be the presence and quantity of bacterium *T forsythia*, and secondary: pain, swelling, trismus, GCF and salivary cytokines, OHRQoL and position and classification of the lower third molars, to avoid bias the measurement of all these outcomes should be standardized.

The bacterium *T forsythia* plays an important role in the development of clinical symptoms of pericoronitis.^[28,29]^ The presence and quantity of *T forsythia* increase in 8 times the risk of developing pericoronitis, when compared to individuals without it.^[28,30,31]^ This bacterium was also associated with the development of pericoronitis in other studies and was present in 40% of their samples.^[32–35]^ Thus, it will be important to measure it properly and quantify this association.^[28]^

PDT causes non-toxic death, leading to destruction of the cell wall and consequent death of the microorganisms. The capacity of PDT to kill bacteria has been studied by several authors; Fontana et al^[16]^ found that bacteria did not reduce to the same degree as has been reported for treatment with antibiotics.

PDT is effective in destroying periodontal pathogens,^[36]^ since most oral bacteria do not absorb the visible light, it is necessary to use the PS, which must act for a period before irradiation.^[37]^

The PS binds to the cell of the microorganism because it needs to bind to negative charges and the membrane of the microorganism has more negative charge than the membrane of the host cell.^[38]^ Gram-negative bacteria induced the dye dimerization more intensely than gram-positive bacteria.^[39]^ The membrane of microorganism are always negatively charged, so the positively charges PS attach and/or aggregate to them and end up interfering on benefits of PDT process.^[40]^

The issue of QoL has largely been neglected within the practice of oral-maxillofacial surgery.^[41]^ OHRQoL is defined as an individual's assessment of how the following elements affect well-being. Magraw et al ^[42]^ concluded that the impact of pain on patients with pericoronitis leads to a worsening OHRQoL, which can influence on the decision of third molar extraction.^[43,44]^ The OHIP-14 questionnaire asks about the adverse impacts of oral conditions on well-being.^[45]^ QoL outcomes may be as important as clinical signs when decisions are rendered regarding third molar extractions.^[26,46]^

For pain measurement, a VAS will be included as the main instrument for assessing the patients’ complaint regarding pain. It is graduated in centimeters, which allows the use of parametric tests, which improves accuracy of data analysis.^[28,47,48]^ Pain affects most domains of QoL,^[49]^ which depends on the extent, duration, acuteness, intensity, affectivity, and meaning of the pain, as well as the underlying disease and the individual's characteristics.^[26]^

In order to avoid bias, and to try to eliminate subjectivity factors of pain and quality of life, the same researcher will measure all outcomes and they should be standardized using established methods as VAS and OHIP-14.

Classifications are used to predict the surgical degree of difficulty, and to evaluate the risk of pre and postoperative complications.^[25]^ Pell and Gregory and Winter techniques,^[26]^ are the most common techniques to evaluate tooth position. It evaluates the tooth depth position in the vertical direction (relative to the occlusal plane and in relation to the neck of the adjacent second molar) and horizontally (as it evaluates the position of the tooth in relation to the ramus of the mandible). In order to classify the position of a tooth, the position of the teeth will be evaluated through panoramic radiography, according to the classification of Pell and Gregory and Winter ^[26]^ performed only by 1 evaluator, following the criteria of imaginary lines as proposed by Almendros-Marques et al.^[27]^

Another researcher will apply the red diode laser. The red light will act to improve tissue repair and decrease the periodontal inflammatory process, especially on facial swelling and trismus ^[22]^ and also will modulate the immunoinflammatory response of the host, interfering in the levels of inflammatory cytokines. Corrêa et al,^[13]^ Candeo et al,^[9]^ and Kellesarian et al^[17]^ found a reduction of the pro-inflammatory cytokines IL1-β, IL-6, IL-8, and TNF-α after the PDT. So we will try to verify if PDT will promote advantageous modulation of cytokine levels in pericoronitis.

Macrophages that are frequently found in pericoronitis are able to produce the wide range of pro-inflammatory and anti-inflammatory mediators, as well as substances that enhance pro and anti-inflammatory substances. These substances act in the development and repair of these lesions, expressing IL-1α, TNF-α, IL-6, TGF-β, and prostaglandins and cytokines that act at the beginning and regulation of inflammatory processes through the activation and differentiation of osteoclasts, activation and proliferation of fibroblasts, production of collagen and neovascularization.^[50]^ Tumor necrosis factor (TNFα) is a cell signaling protein (cytokine) involved in systemic inflammation and is one of the cytokines that make up the acute phase reaction.^[51]^ It is produced chiefly by activated macrophages, although it can be produced by many other cell types. IL1-β, IL-6, and IL-8 are interleukins that act as pro-inflammatory cytokines. IL-10 is an anti-inflammatory cytokine.

The GCF is a transudate of interstitial tissues which is produced by an osmotic gradient, and it is released into the crevicular sulcus. However, during periodontal inflammation, the main mechanism of GCF formation becomes exudative, with an increase in its flow rate, and thus volume.^[52]^ Cytokines produced by various cells are strong local mediators of inflammation.^[53]^ GCF, an exudates, harnessed from the sulcus or periodontal pocket, has been regarded as a promising medium for the detection of periodontal disease activity.^[54]^ GCF has also demonstrated a potential value to evaluate periodontal therapy efficacy.^[23]^

The composition of this fluid resembles that of serum, and the intensity of its flow has been shown to vary as a function of gingival inflammation. There is a wide variation in the methods of collection, storage, and analysis along with elusion protocols making the values alter based on this variation.^[54]^ Kinney et al^[23]^ concluded that the greatest degree of sensitivity was noted with GCF biomarkers compared to saliva biomarkers on the identification of periodontal disease progression, reason why it will be used this method in this study, and it will be compared to salivary cytokines.

MB was chosen as a PS. MB absorbs well the red light^[14]^ and it is the most commonly used PS in dentistry for antimicrobial action.^[6]^ The 3 minutes pre-irradiation time of MB was established based on Joseph et al^[12]^ research. Any decrease of penetration of MB in biofilm may be attributed to a reduced action of PDT,^[16]^ thus an adequate pre-irradiation time is desirable.

It is difficult to predict the efficacy of PS as many factors may contribute to their cellular photoactivity, which is usually related to properties of their monomers species.^[39]^ A decrease in photochemical effects may occur due to dimerization of MB.^[6]^ MB has a tendency to aggregate, also called metachromacy, which is the property of forming dimer or higher aggregation products upon increasing dye concentration or changing the microenvironment (e.g., high ionic strength). It interacts with the anionic macromolecule lipopolysaccharide resulting in the generation of MB dimmers.^[16]^ The absorption spectrum of MB is changed upon aggregation, altering the wavelength of highest absorption to lower wavelengths.^[11]^ Bergmann and Konski^[55]^ showed that the absorption spectrum of monomer and dimer are different. Monomer absorbs light at the wavelength of 660 nm and the dimer at 590 nm. Besides, depending on its ground state monomer/dimmer equilibrium the photochemical properties which can be shifted from type II to type I.^[10,56]^ This fact can interfere with the antimicrobial potential of PDT, since it decreases the formation of singlet oxygen.^[57]^ Here, by changing the MB vehicle, the study aims to verify if the reduced aggregation improves the antimicrobial effect.

Some authors believe that a wavelength of 633 to 670 nm is the best option for laser therapy.^[58,59]^ It is necessary, a wavelength which achieves depth and leads to a triple excitation state of PS.^[7]^ This wavelength will be used and seems to be sufficient for our study.

## Author contributions

**Conceptualization:** Priscila Larcher Longo, Renata Matalon Negreiros, Anna Carolina Ratto Tempestini Horliana, Michelle Palmieri

**Data curation:** Paulo Henrique Braz-Silva, Christiane Pavani, Sandra Kalil Bussadori, Isabel Peixoto Tortamano

**Formal analysis:** Michelle Palmieri, Marcia Pinto Alves Mayer, Paulo Henrique Braz-Silva

**Investigation:** Tânia Oppido Schalch, Renata Matalon Negreiros

**Methodology:** Priscila Larcher Longo, Paulo Henrique Braz-Silva, Edgard Michel-Crosato, Waldyr Antônio Jorge

**Project administration:** Renata Matalon Negreiros, Anna Carolina Ratto Tempestini Horliana

**Resources:** Isabel Peixoto Tortamano, Edgard Michel-Crosato, Christiane Pavani, Marcia Pinto Alves Mayer

**Supervision:** Sandra Kalil Bussadori

**Validation:** Anna Carolina Ratto Tempestini Horliana

**Writing – original draft:** Tânia Oppido Schalch, Renata Matalon Negreiros

**Writing – review & editing:** Priscila Larcher Longo, Renata Matalon Negreiros, Anna Carolina Ratto Tempestini Horliana, Michelle Palmieri, Waldyr Antônio Jorge
